# Global disparities in the detection and management of rheumatic heart disease in pregnancy across endemic settings

**DOI:** 10.3389/fsurg.2026.1838220

**Published:** 2026-07-15

**Authors:** Emili Elkins, Jack Gosden, Guido Ascione, Maximilian Reisinger, Mateusz Kachel, Pavel Overtchouk, Jennifer Haythe, Isaac George

**Affiliations:** 1 Structural Heart and Valve Center, New York Presbyterian Hospital-Columbia University Medical Center, New York, NY, United States; 2 Cardiovascular Research Foundation, New York, NY, United States; 3 University of Vermont Medical Center, Burlington, VT, United States; 4 Center for Cardiovascular Research and Development, American Heart of Poland, Katowice, Poland; 5 Medniscient AI, Dallas, TX, United States; 6 Division of Cardiology, New York Presbyterian Hospital-Columbia University Medical Center, New York, NY, United States

**Keywords:** cardio-obstetrics, echocardiographic screening, mitral stenosis, pregnancy, rheumatic heart disease, valvular disease

## Abstract

**Background:**

Rheumatic heart disease (RHD) remains a leading cause of cardiovascular morbidity and maternal mortality in pregnancy worldwide. Despite near-eradication in high-income countries, RHD continues to disproportionately affect young women in low- and middle-income regions, where delayed diagnosis, limited access to preventive care, and constrained availability of cardiac interventions contribute to adverse maternal and fetal outcomes.

**Objective:**

To present contemporary evidence on the global burden, pathophysiology, detection, surgical and interventional management, of maternal and fetal outcomes of rheumatic heart disease in pregnancy, with principal emphasis on low-resource and endemic settings.

**Methods:**

We conducted a structured narrative review of the existing literature using PubMed/MEDLINE, Embase, Scopus, and the Cochrane Library, identifying observational cohorts, multicenter registries, randomized trials, systematic reviews, international clinical guidelines, and expert reviews published between January 2005 and May 2026. Studies were included if they reported on RHD epidemiology, screening and prophylaxis strategies, cardiac or obstetric outcomes, surgical or transcatheter interventions, valve prosthesis selection, anticoagulation management, or intrapartum and postpartum care during pregnancy. Evidence was narratively synthesized and organized by thematic domains relevant to pregnancy-associated risk.

**Result:**

RHD accounts for the majority of acquired heart disease in pregnancy across endemic regions, with contemporary registries reporting that 30%–70% of cardiac disease complicating pregnancy is rheumatic in origin. Mitral stenosis predominates and is associated with high rates of maternal heart failure, arrhythmia, thromboembolism, and postpartum decompensation. Maternal adverse cardiac events occur in approximately 15%–40% of pregnancies, with mortality ranging from 1%–10% depending on lesion severity, rhythm status, and access to care. Fetal complications, including preterm birth, low birth weight, and pregnancy loss, are common and are exacerbated by severe stenotic disease and prosthetic valves. Screening studies demonstrate that many women with RHD are unaware of their diagnosis prior to pregnancy, while randomized evidence supports echocardiographic screening coupled with secondary antibiotic prophylaxis to reduce disease progression in early-stage RHD. Surgical and interventional management during pregnancy remains high-risk, particularly in women with mechanical heart valves, underscoring the importance of preconception optimization and multidisciplinary care.

**Conclusion:**

Rheumatic heart disease in pregnancy confers substantial and preventable risk to both mother and fetus, particularly in low-resource settings where disease is often advanced at presentation. Improved strategies for early detection, primary and secondary prophylaxis, preconception counseling, and timely referral for valve intervention are essential to reducing maternal and fetal morbidity and mortality. Strengthening health systems to deliver integrated cardio-obstetric care remains central to improving outcomes for women with RHD worldwide.

## Introduction

Rheumatic heart disease (RHD) remains a major contributor to cardiovascular morbidity and maternal mortality in pregnancy worldwide. Despite declines in RHD prevalence across high-income countries, an estimated 33–40 million people are living with RHD globally, with approximately 300,000 deaths annually attributed to complications of the disease (1). Notably, significantly more than half of all RHD cases (22.5 million in 2019) occur in females, with peak prevalence in women aged 25–29 (1). In low- and middle-income regions, RHD is the leading cause of acquired heart disease in pregnant women (2, 3). A recent systematic review in South Asia found that 70% of pregnant women with cardiac disease had underlying rheumatic valvular lesions, punctuating the ubiquity of RHD in maternal cardiac pathology across endemic areas (3). Even in higher-income geographic settings, RHD presents with disproportionate impact on minority populations: a retrospective cohort study of RHD-associated perinatal morbidity and mortality in Australia and New Zealand found that 78% of pregnant RHD patients were Indigenous, with an RHD prevalence of 60 per 10,000 among Aboriginal mothers (4).

Pregnancy imposes hemodynamic stresses that may not be well tolerated in women with rheumatic valve lesions (5, 6). The 30%–50% increase in blood volume, 30% rise in cardiac output, 20% decrease in systemic vascular resistance (SVR), and tachycardia of pregnancy can affect stenotic valve gradients and regurgitant volumes (2, 6).Thus, women with significant rheumatic mitral or aortic valve disease face elevated risks of heart failure, arrhythmias, thromboembolism, and maternal death during pregnancy and puerperium (7). One nationwide confidential enquiry into maternal mortality in South Africa from years 2011–2013 identified complications of RHD (primarily mitral stenosis) as the second-leading cardiac cause of maternal death, attributable to 25% of cardiovascular maternal deaths, second only to cardiomyopathy (6). These deaths were often precipitated by acute heart failure or arrhythmia in late pregnancy or early postpartum, and most were deemed avoidable with improved surveillance and timely intervention (6).

Given the substantial burden and preventable nature of RHD-related maternal complications, optimizing management of affected women before and during pregnancy is critical. However, there remains a paucity in the evidence base to guide care. Historically, management of RHD has relied on observational studies, registry data, and expert consensus to inform treatment rather than randomized trials. In recent years, large multicenter registries such as the Registry of Pregnancy and Cardiac Disease, initiated by the European Society of Cardiology (ESC ROPAC), and systematic reviews have elucidated contemporaneous outcomes and best practices, but significant knowledge gaps persist, thus warranting further discussion (7). In the present review, we consolidate contemporary data on RHD in pregnancy to describe its global burden, pathophysiology, detection, and surgical management, as well as maternal and fetal outcomes.

## Methods

For the present structured narrative review, Embase, PubMed/MEDLINE, Scopus, and the Cochrane Library databases were queried to ascertain relevant studies related to rheumatic heart disease or rheumatic valvular disease in pregnancy, with emphasis on screening, prevention, interventional and surgical management, anticoagulation, delivery planning, postpartum care, and maternal-fetal outcomes. Boolean combinations of the following terms were used: “rheumatic heart disease,” “rheumatic valvular disease,” “valvular disease,” “pregnancy,” “maternal outcomes,” “fetal outcomes,” “cardiac surgery in pregnancy,” “balloon mitral valvuloplasty,” “percutaneous mitral commissurotomy,” “mitral valve repair,” “mechanical valve pregnancy,” “atrial fibrillation,” “anticoagulation in pregnancy,” “delivery,” “postpartum,” and “cardio-obstetrics.” The primary search window was January 1, 2005, through May 20, 2026. Reference lists of relevant articles were further searched to increase the granularity of included studies and to identify guideline documents, landmark registries, and key historical studies needed to contextualize temporal trends.

Eligible sources included observational cohort studies, registries, randomized trials, meta-analyses, systematic reviews, clinical guidelines, and expert reviews published within the search window, with the exception of two ubiquitously referenced observational studies and the 2004 World Health Organization (WHO) Expert Consultation concerning rheumatic fever and RHD for the assessment of temporal trends (8–10). We required that included studies report on RHD epidemiology or RHD in pregnancy and include data on one or more of the following: epidemiology, detection of disease, primary or secondary prophylaxis, hemodynamic and physiologic impacts, cardiac or obstetric outcomes, open surgical or transcatheter interventions, valve prosthesis selection, anticoagulation management, delivery planning, or postpartum management during pregnancy. Studies focusing on congenital or non-rheumatic valvular disease were excluded unless RHD-specific data could be extrapolated.

We then grouped the evidence into thematic categories corresponding to sections of this review. Where appropriate, we reference quantitative outcomes of included studies to illustrate risk magnitude, citing the original sources of such data. Because this manuscript was designed as a structured narrative review rather than a registered systematic review, we did not conduct pooled meta-analysis or formal risk-of-bias scoring. The literature search and selection process is summarized schematically in Supplementary Figure S1.

### Global burden of RHD in pregnancy

RHD remains a disease of significant global burden, especially among young women in low-resource settings (1). The Global Burden of Disease (GBD) 2019 analysis reported approximately 305,000 RHD-related deaths in 2019 alone and over 40.5 million cases of RHD worldwide, representing an increase in absolute case numbers from 33 million cases in 2015, largely due to population growth despite gradual declines in age-standardized mortality rates (1). Critically, RHD has disparate effects on socioeconomically disadvantaged populations; regions with the highest disease prevalence and associated mortality are sub-Saharan Africa, South Asia, the Pacific, and parts of the Middle East, areas where group A streptococcal infections (GAS) are common and access to prophylactic penicillin and echocardiographic screening is limited.

**Figure 1:**
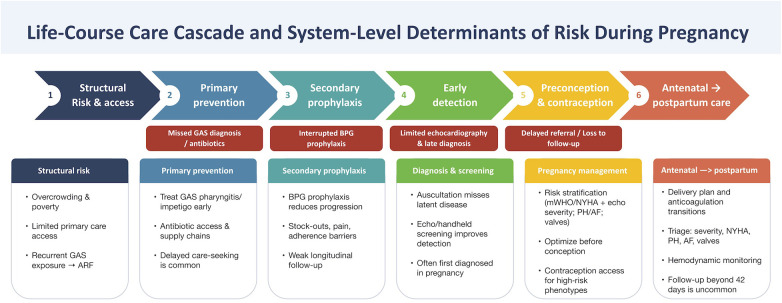
Conceptual overview of the life-course care cascade and system-level determinants of risk during pregnancy in rheumatic heart disease. Multidisciplinary management is explicitly embedded in antenatal and postpartum care because risk reduction depends on coordinated cardiology, maternal-fetal medicine, obstetric anesthesia, nursing, anticoagulation, and referral systems rather than isolated encounters.

Despite near-eradication of RHD in many high-income nations, RHD persists in low- and middle-income countries (LMICs) where upstream determinants such as overcrowding, poverty, limited access to primary healthcare, and delayed recognition of streptococcal disease and acute rheumatic fever remain common (Figure 1). Globally, the largest age-specific prevalence of RHD is in females 25–29 years old, an age coinciding with peak fertility (1). A meta-analysis of heart disease in pregnancy in South Asia, where RHD burden is among the world's highest, found that approximately 70% of all cardiac disease in pregnant women is rheumatic in origin (11). Regional “hotspots” are consistently observed across datasets. Oceania and South Asia repeatedly appear among regions with the highest age-standardized disability-adjusted life-year (DALY) rates in GBD analyses and in review syntheses, while high-income regions demonstrate marked declines and, in some settings, near-elimination of RHD as a common cause of acquired valvular disease (1, 11).

Such findings are not confined to South Asian populations. For example, the 2018 Pan-African Society of Cardiology (PASCAR) position paper reports that RHD accounts for approximately 30% of all cardiovascular disease complicating pregnancy in Africa, further noting an estimated 50% of maternal deaths due to cardiovascular disease occurring beyond 42 days postpartum (12). As the timing of these deaths exceeds the parameters of the conventional 42-day maternal death window as defined by the WHO, this number implies systematic underestimation of true pregnancy-associated cardiovascular mortality. Similarly, the Global Rheumatic Heart Disease Registry (REMEDY) of 3,343 RHD patients in Africa and Asia reported that the majority were female (66.2%) with a median age of 28 years (13). REMEDY also reported marginal contraception use among women of childbearing age, a finding of major pertinence to unintended pregnancy risk in high-risk valvular disease (13). Thus, in regions endemic for rheumatic fever, any woman of reproductive age with known RHD or a history of rheumatic fever must be considered at risk for complications in pregnancy.

Importantly, even within high-income countries, RHD and acute rheumatic fever remain concentrated in marginalized subpopulations. Australian surveillance data demonstrate profound disparities in acute rheumatic fever burden: among 1,425 acute rheumatic fever episodes recorded from 2015 to 2017, 88.8% occurred among Aboriginal and Torres Strait Islander peoples (14). Such subnational heterogeneity matters for pregnancy-focused epidemiology because it predicts where reproductive-age patients with RHD are most likely to reside and where prenatal systems will need screening, referral, anesthetic, cardiology, and obstetric capacity.

Beyond modeled burden, hospital-based cardiovascular syndromic data provide a second lens on severity and case fatality in endemic regions. The Sub-Saharan Africa Survey of Heart Failure (THESUS-HF) registry, which enrolled adults hospitalized with acute heart failure across multiple countries, identified rheumatic heart disease as a major etiology, accounting for 14.3% of acute heart failure presentations (15). In-hospital mortality was 4.2%, and 180-day mortality was 17.8%, emphasizing the high short- and medium-term risk associated with advanced structural disease in settings where timely definitive interventions are often unavailable (15). Although THESUS-HF is not pregnancy-specific, its findings are highly relevant for pregnancy epidemiology in LMICs because pregnancy can reveal previously compensated valvular disease, and because maternal decompensation commonly occurs where baseline disease is advanced and access to specialized cardiovascular care is limited (15).

### Pathophysiology

RHD arises as a delayed autoimmune sequela of acute rheumatic fever (ARF) following untreated or inadequately treated group A beta-hemolytic Streptococcus pharyngitis (16–18). In endemic settings, however, there is increasing evidence that GAS skin infection may also be an important antecedent to RHD (17). ARF is a delayed, non-suppurative inflammatory syndrome that typically develops after a 1–5-week latency period following infection and may involve multiple organ systems (16, 18). While high-income regions have observed decrements in overall ARF incidence, RHD persists as a principal contributor of morbidity and mortality worldwide, with disproportionate impact among children and young adults in LMICs (16, 19). While the pathophysiology of ARF is not completely understood, it is thought to involve molecular mimicry and cross-reactive humoral and cellular immune responses to streptococcal antigens in a genetically susceptible host (16, 20, 21). ARF is diagnosed clinically using the Jones criteria; importantly, the 2015 revision incorporates population risk and Doppler echocardiography to improve detection of carditis, including subclinical disease in endemic areas (18, 22).

Cardiac involvement in ARF is characterized by pancarditis, affecting the endocardium, myocardium, and pericardium (16). Endocardial inflammation manifests as valvulitis with small, sterile fibrinous vegetations along the lines of valve closure, most commonly involving the mitral valve (16, 23). Myocardial involvement is marked by the formation of Aschoff bodies, pathognomonic granulomatous lesions consisting of central fibrinoid necrosis surrounded by lymphocytes, plasma cells, and activated macrophages known as Anitschkow cells (16, 20, 24). Pericardial involvement, when present, characteristically produces a transient fibrinous pericarditis (16, 20, 24). Importantly, while myocardial inflammation may contribute to acute hemodynamic compromise, endocardial and valvular injury drives long-term morbidity (16).

While extracardiac ARF manifestations generally resolve, valve injury may persist and progress with recurrent GAS infections and repeated inflammatory episodes, leading to fibrosis, commissural fusion, and chordal thickening or shortening characteristic of chronic RHD (16, 23). The mitral valve is most frequently involved and may evolve to mixed regurgitation and stenosis; advanced mitral stenosis produces the classic narrowed “fish-mouth” or “buttonhole” orifice (16, 23). Elevated left atrial pressure leads to left atrial dilation, predisposing to atrial fibrillation and thromboembolism, while chronic pulmonary venous hypertension may progress to pulmonary arterial hypertension and right-sided heart failure (16). Aortic valve involvement is commonly observed in combination with mitral disease, with early regurgitation due to leaflet retraction and later fibrotic or calcific stenosis; tricuspid valve dysfunction is less often primary rheumatic disease and is frequently functional in the setting of pulmonary hypertension (16, 23).

Pregnancy-specific physiology magnifies these anatomic lesions through different mechanisms in stenotic and regurgitant disease. Stenotic lesions are particularly vulnerable because the valve orifice is fixed and the transvalvular gradient is flow dependent. As plasma volume and cardiac output rise, even modest increases in diastolic flow across a stenotic mitral valve can produce disproportionate increases in left atrial pressure and pulmonary venous congestion. Tachycardia further shortens diastole, reducing filling time and accentuating the gradient across mitral stenosis. This is why severe rheumatic mitral stenosis can convert from compensated disease to pulmonary edema, atrial fibrillation, or right-sided failure during late pregnancy, labor, or the early postpartum period. Regurgitant lesions are often better tolerated because the fall in SVR may facilitate forward flow; however, the expanded intravascular volume, tachycardia, and increased total stroke volume of pregnancy can also increase regurgitant volumes when leaflet malcoaptation, annular dilation, or chordal restriction is substantial. Thus, women with severe mitral or aortic regurgitation remain at risk of ventricular dilation, congestion, and arrhythmia, especially when baseline ventricular function is impaired or when anemia, preeclampsia, infection, or poor rate control supervenes (2, 6, 7).

Clinically important complications of established RHD include atrial fibrillation, heart failure, thromboembolic events, and infective endocarditis; pregnancy can precipitate cardiovascular decompensation, particularly in patients with severe stenotic lesions and pulmonary hypertension (16, 19, 25). Given that the inciting GAS infection is preventable and disease progression is modifiable with secondary antibiotic prophylaxis, most commonly intramuscular benzathine penicillin G, RHD remains a paradigmatic example of infection-triggered, immune-mediated cardiovascular disease (16, 26, 27).

### RHD primary prophylaxis and screening in endemic low-resource settings

Primary prevention of RHD hinges on interrupting the causal chain that begins with GAS infection and culminates in ARF and chronic valvular damage. In practice, this agenda has two complementary components in endemic LMICs: primary prophylaxis, defined as timely antibiotic therapy for GAS infection, classically pharyngitis but increasingly recognized to include skin infection in some endemic settings; and echocardiographic screening, intended to identify latent or early RHD so that secondary antibiotic prophylaxis and longitudinal surveillance can be initiated before advanced valve disease develops. Contemporary valvular heart disease guidelines continue to emphasize rapid detection and treatment of streptococcal pharyngitis as the mainstay of primary prevention (28–30).

Globally, GAS pharyngitis is exceedingly common, with over 600 million cases in children per year, leading to approximately 460,000 new ARF cases annually (31). Effective antibiotic therapy with a single intramuscular dose of benzathine penicillin G (BPG) is the mainstay for suspected or confirmed GAS pharyngitis, as well as superficial skin infections in settings where these are linked to ARF (17, 18). One historical systematic review and meta-analysis of randomized or quasi-randomized trials of antibiotics for sore throat identified 10 eligible trials (*n* = 7,665), with pooled estimates demonstrating a 70% relative reduction in ARF with antibiotics (RR 0.32; 95% CI 0.21–0.48), corresponding to an absolute risk reduction of 1.67% and a number needed to treat of 53 (32). Restricting to trials evaluating penicillin, the estimated protective effect increased (RR 0.20; 95% CI 0.11–0.36; number needed to treat 60) (32).

BPG is inexpensive and is listed on the WHO Essential Medicines List (33). However, practical challenges severely limit its utilization for primary and secondary prevention in LMICs, with providers reporting recurrent shortages, stockouts, and concerns about product quality (34–36). A 2011 World Heart Federation survey of healthcare providers across 24 countries in Africa, Asia-Pacific, and the Americas found that “minimal access to BPG” was a widespread problem; among 39 surveyed clinicians, more than one-third reported that their available BPG supply was insufficient to provide prophylaxis for all eligible patients according to guideline-recommended schedules (35, 36). More recent evidence indicates that these access constraints have not resolved. A 2024 global supply analysis described BPG as a globally indispensable medicine for syphilis, GAS infection, ARF, and RHD, and attributed recurring shortages to a fragile market characterized by low profit margins, limited demand visibility, and a reduced number of active pharmaceutical ingredient manufacturers (37). The WHO 2025 fact sheet likewise emphasizes that a steady supply of quality-assured BPG is an essential prerequisite for treatment of sore throat and prevention of recurrent infection, while noting that the antibiotic remains prone to global shortages (38). Thus, the historical WHF survey should be interpreted as an early marker of a persistent supply-chain vulnerability rather than as an isolated finding.

Meta-analysis of historical data indicates roughly 1 case of rheumatic fever is averted per 50–60 patients treated for suspected GAS pharyngitis (32). With reference to the high medical costs of managing rheumatic heart disease, with frequently reported values in the tens of millions of dollars at the population level, primary prevention via antibiotics is extremely cost-effective (39). School-based sore-throat clinics and antibiotic therapy in New Zealand markedly reduced ARF incidence in high-risk Māori and Pacific Islander children (40, 41). Similar penicillin prophylaxis initiatives in Pacific Island nations and parts of Africa are underway, but scale-up remains hampered by infrastructure and sustainability issues (42).

Numerous studies in high-risk cohorts emphasize the consequences of failing to implement primary prophylaxis. In a recent retrospective cohort study of children diagnosed with RHD on echocardiographic screening, 90% had no prior history of ARF, yet virtually all had experienced multiple GAS infections, predominantly untreated impetigo or “skin sores,” in the years before RHD diagnosis (43). Principal barriers include low health literacy about the link between a “simple” sore throat and rheumatic fever, limited access to medical evaluation in rural areas, and diagnostic uncertainty (42, 44).

The principal constraints on primary prophylaxis in endemic regions are rarely conceptual. Rather, they relate to case identification and feasibility of treatment at scale. A throat swab bacterial culture is the diagnostic gold standard for confirming GAS pharyngitis, and rapid antigen detection tests are valuable near-patient diagnostic tools (8). Unfortunately, both culture and rapid antigen detection tests are often unavailable or too costly in many LMIC settings. Thus, where laboratory confirmation of GAS is not feasible, clinicians rely on symptom-based algorithms for treatment decisions. One historical analysis in a pediatric urban-Egypt cohort evaluated the WHO acute respiratory infection guideline for sore throat and found it to be highly specific but only approximately 12% sensitive, failing to capture 88% of true GAS cases (9). By modifying the criteria to treat if either tonsillar exudate or enlarged cervical node is present, sensitivity rose to 84% at the cost of specificity dropping to approximately 40% (9).

Because primary prophylaxis is variably implemented and ARF is often clinically silent or retrospectively unrecognized, echocardiographic screening has been pursued as a strategy to detect disease at a stage when intervention is still preventive rather than salvage. The justification for screening rests on the substantial reservoir of latent disease that is not detectable by physical examination and that may progress to clinically significant valve lesions over time.

### Global yield of echocardiographic screening

A large contemporary meta-analysis of population-based echocardiographic studies by Noubiap and colleagues explicates both the scale of latent disease and the sensitivity gap between auscultation and echocardiography (45). Across 82 studies comprising 1,090,792 participants, the pooled prevalence of RHD was 26.1 per 1,000 (95% CI 19.2–33.1) in studies using the World Heart Federation (WHF) criteria, compared with 11.3 per 1,000 (95% CI 7.2–16.2) using older WHO criteria (45). This analysis also found a substantial effect of screening strategy: the pooled prevalence estimate was 6.4 per 1,000 (95% CI 4.0–9.2) for an auscultation-first strategy vs. 21.2 per 1,000 (95% CI 15.3–28.1) for an echo-first strategy, consistent with the conclusion that reliance on auscultation alone identifies only a minority of echocardiographically detectable disease (45).

In a 24-month prospective longitudinal cohort study evaluating echocardiographic screening of pregnant women in three Ugandan low-resource health centers, Beaton and colleagues reported that 97% of women found to have heart disease on screening were previously unaware of their diagnosis, supporting the concept that opportunistic or population-based screening can uncover clinically important disease that is not captured by routine clinical pathways in resource-limited settings (46).

Performance limitations of auscultation are not merely theoretical. A WHO systematic review evaluating routine antenatal echocardiography in endemic areas summarized studies demonstrating missed diagnoses when screening relies solely on clinical examination (47). In one report included in the review, auscultation detected murmurs in only 6 of 27 cases of subclinical RHD identified by echocardiography (48). The WHO-guideline review also reveals a key paucity in the literature informing policy decisions: no studies directly compared routine antenatal echocardiographic screening vs. standard care in endemic regions, meaning that policy decisions about universal antenatal echocardiography require extrapolation from observational feasibility studies and from evidence conferred by studies in school-age screening cohorts (47).

### Contemporary diagnostic frameworks: WHF 2023 guidelines and task-sharing adaptations

The expansion of screening programs is linked to standardization of diagnostic definitions, with the 2023 WHF guidelines representing a shift from an earlier “borderline” and “definite” disease categorization toward a stage-based continuum integrating risk assessment of disease progression (30). The WHF guidelines explicitly introduce two sets of echocardiographic criteria: screening criteria intended for rapid detection in high-prevalence settings, including task-sharing and handheld devices, and confirmatory criteria for expert use in establishing diagnosis (30). The guideline notes that screening criteria are designed to increase sensitivity at the expense of specificity and recommends a two-step strategy, screening followed by confirmatory study, where feasible. These guidelines further offer a pragmatic innovation in their explicit accommodation of handheld echocardiography by simplifying certain Doppler requirements and providing operational thresholds to balance false positives and false negatives (30).

### Screening linked to prophylaxis: trial evidence in endemic settings

Although screening has been widely implemented in research and programmatic contexts, high-quality evidence linking screening to clinically meaningful outcome improvement has historically been limited. In the last decade, however, randomized evidence has emerged in exactly the populations most relevant to LMIC prevention strategies. Results from the GwokO Adunu pa Lutino (GOAL) trial, the first randomized trial to assess whether regular intramuscular BPG can modify the trajectory of screen-detected latent RHD, provide one of the strongest proofs of concept supporting screening coupled with secondary prophylaxis (49). Investigators screened 102,200 children, identified 3,327 with latent RHD for further evaluation, and randomized 916 eligible participants (49). Among 818 participants in the modified intention-to-treat analysis, progression of latent RHD occurred in 3 children (0.8%) assigned to prophylaxis vs. 33 children (8.2%) assigned to control, yielding a risk difference of −7.5 percentage points (95% CI −10.2 to −4.7; *P* < 0.001) (49). Such results are consistent with the WHF 2023 guideline statement that randomized evidence supports secondary antibiotic prophylaxis to reduce progression risk in early-stage RHD in children aged 5–17 years (30).

Karki and colleagues conducted systematic echocardiographic screening in Nepal through a cluster-randomized trial involving 17 schools and 12,048 children, with two years of screening followed by a four-year follow-up (50). In the primary follow-up analysis, there were 17 RHD cases (3.8 per 1,000) in experimental schools compared with 51 cases (10.8 per 1,000) in control schools, resulting in an odds ratio of 0.34 (95% CI 0.11–1.07; *P* = 0.06) (50). Secondary analyses indicated reductions over time within the screened clusters, with a baseline to follow-up odds ratio of 0.29 for definite RHD and 0.52 for borderline RHD (50). A later cross-sectional study by Chillo and colleagues provides additional evidence on programmatic feasibility and expected yield when screening is integrated into school health initiatives (51). In a Tanzanian school-based RHD prevention program, handheld echocardiography identified subclinical RHD in 95 children, corresponding to a prevalence of 2.1% (95% CI 1.7–2.6), including 59 definite and 36 borderline cases, in a screened sample of 4,436 participants (51).

The Nepal trial is instructive for two principal reasons with reference to RHD prevention (50). Firstly, it demonstrates the feasibility of implementing large-scale school screening in an endemic setting, with longitudinal follow-up. Secondly, it emphasizes that measurable population-level benefits may be contingent upon pragmatic implementation factors, including the adoption of confirmatory imaging, initiation and adherence to prophylactic measures, and the robustness of follow-up systems (50). Although cross-sectional prevalence studies cannot definitively establish outcome benefits, the Tanzanian experience underscores the magnitude of the latent pool detectable within endemic school populations and endorses the WHF emphasis on task-sharing and the utilization of handheld imaging as a feasible approach to augment detection in regions where expert echocardiographic services are limited (30, 51).

### Adverse maternal outcomes

Hospital- and registry-based data from endemic regions show that major maternal cardiac events occur in a clinically significant proportion of pregnancies complicated by RHD, with the precise incidence varying by case mix, severity distribution, referral bias, and outcome definitions. In LMIC settings, RHD in pregnancy contributes significantly to maternal morbidity and mortality. In South Africa, for example, a confidential audit of maternal deaths associated with cardiovascular disease across 2011–2013 found complications of RHD associated with 38 reported deaths, primarily attributable to mitral valve stenosis (6). Many of these women died in late pregnancy or soon postpartum from acute pulmonary edema, arrhythmias, or embolic strokes due to their valvular disease (6). Notably, the audit found that 22.9% of clinician assessors believed that delays in patient referral to an appropriate level of care contributed to death (6).

In the Madras Medical College Pregnancy and Cardiac (M-PAC) Registry from India, which prospectively enrolled 1,005 women with 1,029 consecutive pregnancies from July 2016 through December 2019, RHD accounted for 42% of diagnoses (433/1,029), underscoring its dominance within LMIC cardio-obstetric practice (52). In the overall cohort, major cardiac events occurred in 15.2% and maternal mortality was 1.9%, with the highest mortality observed among women with prosthetic heart valves (8.6%) (52). Importantly, deaths in this registry clustered after delivery: 15 of 20 maternal deaths occurred in the postpartum period (52). The distribution of major cardiac event subtypes reinforces the central role of decompensation physiology: heart failure was the most common event, followed by arrhythmic complications, thromboembolic events, and hemorrhagic events (52).

RHD-specific cohorts provide more granular estimates. In a large prospective cohort of pregnancies with valvular rheumatic heart disease managed at a tertiary center in south India (820 pregnancies, 2011–2018), composite adverse cardiac outcome occurred in 14.9%, with 12 maternal deaths (1.5%) (53). Cardiac morbidity was again dominated by heart failure (13.5%), followed by arrhythmias (6.7%), thromboembolic events (4.1%), and bleeding complications (2.7%) (53).

Lesion severity markedly influences maternal risk. In the 2018 ROPAC analysis by van Hagen and colleagues, which concentrated on rheumatic mitral valve disease (*n* = 390), outcomes varied between mitral stenosis and isolated mitral regurgitation cohorts (7). Heart failure was observed in 31.1% of pregnancies within the mitral stenosis group, compared with 16.2% in the mitral regurgitation group (7). This signal strengthened with increasing severity of stenosis: heart failure was reported in 5.7% of mild mitral stenosis, 28.2% of moderate mitral stenosis, and 50.0% of severe mitral stenosis (7). Maternal mortality in this cohort was approximately 1% (7). Collectively, these data exemplify a fundamental principle concerning RHD in pregnancy: stenotic lesions, particularly severe mitral stenosis, are disproportionately linked to acute hemodynamic deterioration (7).

The broader consequence of late diagnosis and limited access to longitudinal care is further reflected in maternity cohorts from resource-limited environments. In a South African low-resource cohort of pregnant women referred to a dedicated Cardiac Disease and Maternity Clinic, valvular heart disease comprised 26.2% of the high-risk cardiac subgroup, and valvular disease accounted for 42.1% of maternal deaths within that high-risk cohort (54). While “valvular heart disease” is not synonymous with rheumatic disease in all settings, in many LMIC cohorts, valvular disease is predominantly rheumatic, and these data reinforce that valve pathology is a leading pathway to maternal death where surveillance and timely intervention are constrained.

The feasibility and availability of multidisciplinary care is therefore not incidental to outcomes. It determines whether women can access cardiology review, serial echocardiography, anesthesia planning, anticoagulation monitoring, timely escalation, and delivery in a facility capable of managing acute pulmonary edema, arrhythmia, hemorrhage, or valve thrombosis. This is illustrated by an observational study from the Top End of Australia's Northern Territory, where Lam and colleagues evaluated 129 pregnancies with RHD over a 9.5-year period at the region's largest obstetric referral hospital (55). All women were Aboriginal or Torres Strait Islander, 85% had RHD priority level 2 or 3, and obstetric, cardiac, and anesthetic data were explicitly evaluated (55). Despite the social, geographic, and cultural complexity of the cohort, there were no maternal or neonatal deaths, and only 1 of 28 emergency cesarean deliveries was undertaken for cardiac reasons (55). The authors attributed these favorable outcomes to care in a multidisciplinary center with substantial experience managing the medical and cultural complexities of RHD in pregnancy (55). This experience is important because it demonstrates that multidisciplinary care is feasible and impactful even in a remote high-burden setting, while also underscoring that such care requires deliberate infrastructure rather than passive referral. In many endemic regions, the absence of on-site cardiology, echocardiography, subspeciality anesthesia care (e.g., obstetric and cardiac anesthesia), blood products, intensive care, and valve intervention services remains a principal mechanism by which otherwise manageable disease becomes fatal.

### Interventional and surgical management during pregnancy

Management of RHD in pregnancy begins with preconception optimization whenever possible. Women with moderate or severe valvular disease require lesion-specific risk stratification, echocardiographic assessment of valve area and gradients, evaluation of pulmonary pressure and ventricular function, rhythm assessment, and counseling regarding the maternal and fetal implications of pregnancy (28, 29, 56). Intervention before conception is preferred for symptomatic severe valve disease or lesions likely to become poorly tolerated during pregnancy, because emergency intervention during pregnancy places both mother and fetus at risk. This is particularly true for rheumatic mitral stenosis, where pregnancy can rapidly increase transmitral gradients and pulmonary pressures.

For severe symptomatic rheumatic mitral stenosis during pregnancy, medical therapy remains the initial approach. Restricted activity, careful volume management, beta-blockade to prolong diastolic filling, and diuretics for persistent congestion are core measures (56). If severe symptoms or pulmonary hypertension persist despite medical therapy, percutaneous balloon mitral valvotomy (PBMV), also termed percutaneous mitral commissurotomy, should be considered when valve anatomy is favorable and left atrial thrombus or more-than-mild mitral regurgitation are absent (2, 56). PBMV directly addresses the pregnancy-specific pathophysiology of rheumatic mitral stenosis by enlarging the valve orifice, reducing the transmitral gradient, lowering left atrial pressure, and reducing pulmonary venous congestion. In experienced hands, it can avert recurrent pulmonary edema and permit continuation of pregnancy while avoiding cardiopulmonary bypass (2, 56). Contemporary guideline recommendations favor pre-pregnancy intervention for women with mitral stenosis and valve area <1.5 cm2, and during pregnancy recommend considering PBMV for severe symptoms or systolic pulmonary artery pressure >50 mmHg despite medical therapy (56).

The low-resource implication is two-fold. First, PBMV is among the few definitive cardiac interventions that can be performed during pregnancy without cardiopulmonary bypass, making it especially valuable in endemic settings where rheumatic mitral stenosis is common. Second, access is uneven: PBMV requires echocardiographic expertise, fluoroscopy or echocardiography-guided procedural skill, radiation-minimization protocols, and capacity for surgical rescue if complications occur. Where PBMV is unavailable, women may be managed medically until delivery or referred across long distances, both of which can be hazardous when pulmonary edema, atrial fibrillation, or pulmonary hypertension are present. Open valve surgery during pregnancy is therefore reserved for situations in which maternal mortality risk is high and other treatment options have failed (56).

Predominantly regurgitant rheumatic lesions require different management logic. Because chronic mitral or aortic regurgitation may be better tolerated during pregnancy than stenosis, medical management with diuretics for congestion and afterload-sensitive supportive care is often sufficient when ventricular function is preserved (56). However, severe rheumatic mitral regurgitation with symptoms, impaired ventricular function, or marked ventricular dilation should ideally be addressed before pregnancy (56). In this specific cohort, mitral valve repair deserves explicit consideration. Valve repair avoids prosthesis-related anticoagulation and may be particularly attractive for women desiring pregnancy, but rheumatic pathology often involves leaflet thickening, retraction, commissural fusion, and chordal shortening that can limit repair durability (57). Contemporary RHD surgical reviews emphasize that repair is preferable over replacement for rheumatic mitral regurgitation when a durable repair is achievable, but that repair expertise and follow-up capacity are not available to the vast majority of patients in endemic regions (57). Thus, the decision between repair and replacement should be individualized, should involve surgeons experienced in rheumatic repair when possible, and should be situated within the patient's reproductive goals, access to anticoagulation monitoring, and likelihood of durable follow-up.

During pregnancy, mitral valve repair or replacement for regurgitant lesions is rarely undertaken unless maternal status is refractory and life-threatening. Cardiopulmonary bypass is associated with fetal risk, and fetal loss has historically been substantial when open cardiac surgery is required during pregnancy. Therefore, the highest-yield management pathway is preconception identification of severe regurgitant rheumatic disease, timely referral for repair or replacement before pregnancy when indicated, and coordinated counseling regarding valve choice. In many young women with RHD, the valve substitute decision is inseparable from future pregnancy risk: mechanical valves offer durability but require complex anticoagulation during pregnancy, while bioprosthetic valves avoid long-term anticoagulation but degenerate more rapidly in young patients, potentially requiring reintervention (56, 57).

### Prosthetic valves and anticoagulation: high maternal risk and competing fetal hazards

A clinically significant subset of women in endemic areas enter pregnancy after valve surgery for rheumatic disease, including mechanical prostheses. Such phenotypes introduce competing maternal and fetal hazards: insufficient anticoagulation raises the risk of valve thrombosis and embolism, while fetal-safer anticoagulation strategies can be logistically difficult in low-resource contexts. The problem is most acute for mechanical mitral valves, where thrombogenicity is high and where many patients require lifelong anticoagulation from adolescence or young adulthood.

Contemporary guideline syntheses highlight persistently poor outcomes for mechanical valves, with limited improvement over time in international registries. In the 2025 ESC guideline synthesis for the management of cardiovascular disease and pregnancy, no significant improvement in the proportion of event-free pregnancies with a live birth in women with mechanical heart valves across the initial ROPAC II analysis and the later ROPAC III was observed (58% vs. 54%, respectively) (56, 58). Across these datasets, thrombotic complications are reported in approximately 9%–24% of pregnancies, while major bleeding complications occur in roughly 20%–30%, substantiating the intrinsic high-risk nature of mechanical valve pregnancy even in contemporary practice and high-resource settings (56, 58). The same guideline discussion cites data from the M-PAC registry prosthetic-valve subgroup, describing high fetal death and major adverse cardiovascular event rates in a context where underlying valvular disease is more often rheumatic and where system-level constraints may limit anticoagulation monitoring and timely intervention (52, 56).

Anticoagulation during pregnancy must be differentiated by indication. For women with rheumatic mitral stenosis complicated by atrial fibrillation, left atrial thrombus, or prior embolism, full therapeutic-dose anticoagulation is recommended (56, 59). Atrial fibrillation is particularly consequential in rheumatic mitral stenosis because loss of atrial contribution, rapid ventricular response, and shortened diastole all worsen left atrial pressure and pulmonary congestion while increasing thromboembolic risk. In a contemporary propensity score-matched analysis, RHD pregnancies complicated by atrial fibrillation experienced higher adverse cardiac event rates than those without atrial fibrillation, reinforcing that rhythm status is not simply a marker of disease severity but a management-defining risk factor (59).

In women with mechanical valves, no anticoagulation regimen is risk-free. Continuous vitamin K antagonist therapy is generally the most effective strategy for preventing maternal valve thrombosis but crosses the placenta and introduces dose-related risks of embryopathy, fetal loss, and fetal bleeding (56, 58). Low-molecular-weight heparin avoids placental transfer but requires strict dosing and reliable anti-factor Xa monitoring; without such monitoring, maternal valve thrombosis risk may be unacceptable (56, 58). Unfractionated heparin has a role near delivery or when rapid reversal is required, but maintaining therapeutic anticoagulation can be difficult and may require inpatient management. These competing risks are magnified in LMICs where anti-factor Xa testing, INR monitoring, medication supply, emergency echocardiography, thrombolysis, cardiac surgery, and intensive care may be inconsistently available.

Practical anticoagulation management therefore requires pre-pregnancy counseling and a written plan that specifies the regimen by gestational age, laboratory monitoring schedule, thresholds for dose adjustment, timing of transition before delivery, and postpartum restart strategy (56, 60, 61). For planned delivery, anticoagulation timing should be coordinated with obstetrics and anesthesiology because neuraxial anesthesia safety depends on the drug, dose, and time since last administration (60). Postpartum anticoagulation is equally critical: the early puerperium combines hypercoagulability, rapid hemodynamic shifts, bleeding risk, and frequent interruptions of anticoagulation around delivery. In women with prosthetic valves, a failure to coordinate anticoagulation restart can produce catastrophic valve thrombosis, while overly aggressive restart may worsen postpartum hemorrhage. This is the clinical context in which a pregnancy heart team is not merely ideal but essential.

### Delivery, postpartum, and multidisciplinary management

The postpartum period is consistently identified as a high-risk window for women with RHD, yet risk begins before delivery. Labor combines pain, catecholamine release, tachycardia, autotransfusion with uterine contractions, fluid shifts, hemorrhage risk, and abrupt changes in preload and afterload. For women with mitral stenosis, these changes can rapidly increase pulmonary venous pressures; for women with regurgitant lesions or ventricular dysfunction, they can precipitate congestion or arrhythmia. Accordingly, delivery planning should be explicit and individualized, ideally completed in the late second or early third trimester for women with moderate or severe disease. The plan should specify location of delivery, mode and timing, intrapartum monitoring, anesthesia strategy, anticoagulation transition, postpartum surveillance level, and contingency pathways for pulmonary edema, atrial fibrillation, hemorrhage, thromboembolism, or urgent valve intervention (56, 60, 61).

Vaginal delivery is preferred for most stable patients with cardiovascular disease because it is associated with less bleeding, thrombosis, infection, and abrupt physiologic stress than cesarean delivery (60, 61). Cesarean delivery should generally be reserved for obstetric indications or specific cardiac indications such as severe decompensation, inability to safely manage anticoagulation, aortic or other lesions for which labor is judged unsafe, or severe disease in which controlled timing and personnel availability are essential (56, 60, 61). In women with significant mitral stenosis, the goals are to avoid tachycardia, hypoxia, anemia, pain-driven sympathetic surges, and fluid overload. Early neuraxial labor analgesia can blunt sympathetic stimulation, while assisted second stage may be considered to reduce prolonged Valsalva and hemodynamic burden (60). For severe lesions, pulmonary hypertension, prosthetic valves, or recent decompensation, delivery should occur in a center with cardiology, maternal-fetal medicine, obstetric and cardiac anesthesia, critical care, blood bank support, and access to advanced cardiovascular therapies or transfer pathways (56, 60, 61).

Hemodynamic monitoring should be calibrated to lesion severity. Routine telemetry, pulse oximetry, strict fluid balance, avoidance of unnecessary intravenous fluids, and early use of diuretics when congestion develops are appropriate for many high-risk RHD patients. Invasive arterial monitoring, central access, or intensive care admission may be appropriate for women with severe mitral stenosis, pulmonary hypertension, ventricular dysfunction, mechanical valves with complex anticoagulation transitions, or recent heart failure. However, monitoring is only useful when paired with personnel capable of interpreting and responding to the data. This is another reason that access to cardiology and anesthesiology is central to safe care rather than an ancillary feature.

The immediate postpartum interval deserves special emphasis. Autotransfusion from uterine involution, mobilization of extravascular fluid, and relief of caval compression can abruptly increase preload, while hemorrhage, anemia, infection, and pain can destabilize patients with limited cardiac reserve. The first 24–48 h after delivery are particularly important for monitoring of heart failure and arrhythmia, and many women with severe disease warrant extended observation beyond routine obstetric discharge windows (52, 56, 60). Postpartum care should include reassessment of volume status, rhythm monitoring when indicated, resumption or adjustment of anticoagulation, lactation-compatible cardiovascular medications, contraception counseling, continuation of secondary prophylaxis, and linkage back to RHD surveillance and valve intervention pathways.

### Community-Level experience in Low-resource settings: outcomes when diagnosis is late and resources are limited

**Figure 2:**
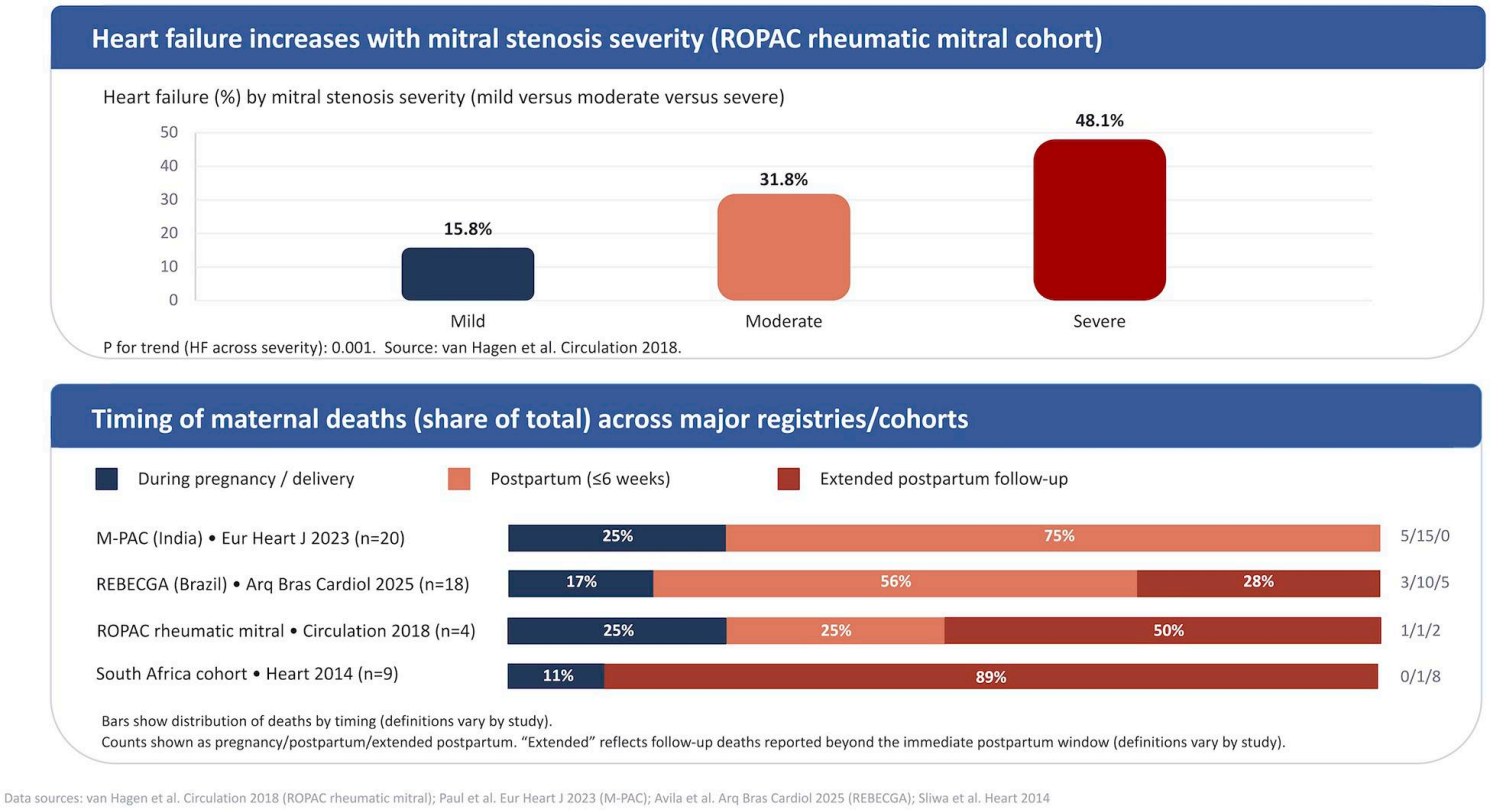
Registry-derived risk signals in rheumatic and valvular heart disease during pregnancy. Heart failure increases with rheumatic mitral stenosis severity in the ROPAC rheumatic mitral cohort. Across selected registries and cohorts, maternal deaths frequently occurred after delivery, underscoring the need for postpartum surveillance. Timing categories vary by study and are shown descriptively rather than as pooled estimates.

Outcomes observed in community-linked studies aid in contextualizing registry estimates from tertiary centers, which may fail to capture the most severely ill women who never access specialist care. In a low-resource Ugandan cohort study, echocardiographic screening identified cardiovascular disease in 17 per 1,000 pregnancies, with RHD accounting for 15 per 1,000 pregnancies and representing 88% of identified cardiovascular disease (46). Cardiovascular complications occurred in 51% of women with heart disease, most commonly heart failure. Maternal mortality in women with heart disease was 10.7% (6/56) compared with 0.2% (3/1,409) in women without heart disease, and neonatal mortality was 5.4% compared with 2.5% (46) (Figure 2).

Regional evidence from Latin America further reinforces the centrality of rheumatic valvular pathology in adverse pregnancy outcomes where RHD persists. In the Brazilian Registry of Heart Disease and Pregnancy (REBECGA; *n* = 638), valvular heart disease was the most common diagnostic category (37.8%) (62). Complications in the overall cohort included heart failure in 16.7% and arrhythmias in 10.7%. Maternal mortality was 2.8% (18 deaths), with most deaths occurring postpartum (10 of 18). Fetal losses occurred in 3.2%, and preterm births in 25.7% (62). Within the valvular subgroup, rheumatic etiology accounted for 69.7% (168 patients). In the rheumatic subgroup, the major cardiac complication rate was 42.9%, and maternal mortality was 3.9% (62).

These community and registry data converge on a common theme: late diagnosis and limited resources do not merely delay care, they reshape prognosis. Women with undiagnosed RHD may enter pregnancy without lesion staging, rhythm assessment, secondary prophylaxis, contraception counseling, or preconception intervention. Once pregnant, access barriers can prevent referral until pulmonary edema, atrial fibrillation, thromboembolism, or advanced heart failure has already occurred. In such environments, the highest-impact interventions may be system-level: integration of RHD screening into antenatal care where prevalence justifies it, reliable BPG supply, locally available echocardiography, standardized referral thresholds, multidisciplinary delivery pathways, and postpartum follow-up that extends beyond the conventional obstetric window.

## Conclusions

Rheumatic heart disease in pregnancy remains a persistent and preventable contributor to maternal and perinatal morbidity in many low- and middle-income countries. The evidence presented in the present review highlights a consistent pattern across diverse endemic settings in which delayed diagnosis, limited access to specialized cardio-obstetric care, and health system infrastructure fragmentation continue to drive avertible complications. Contemporary data from hospital cohorts, national registries, and international collaborations demonstrate that maternal cardiac events, particularly heart failure and arrhythmias, occur in a substantial proportion of pregnancies complicated by RHD, with the highest risk concentrated among women with severe valvular lesions, especially mitral stenosis.

At the population level, recent randomized and programmatic studies linking echocardiographic screening to antibiotic prophylaxis provide strong proof of concept that earlier detection and secondary prevention can meaningfully alter the native concatenation of latent disease. At the clinical level, severe rheumatic mitral stenosis remains the lesion in which pregnancy physiology is most hazardous and in which timely PBMV may be pregnancy preserving when medical therapy fails and anatomy is suitable. For predominantly regurgitant lesions, the crucial management opportunity is often preconception, when durable mitral valve repair or other definitive intervention can be considered before pregnancy magnifies hemodynamic stress. For women with mechanical valves or atrial fibrillation, anticoagulation management must be individualized and tightly coordinated across antepartum, delivery, and postpartum intervals. Translating such findings into large-scale impact will require sustained investments in health system capacity, including workforce training, diagnostic infrastructure, reliable penicillin supply chains, anticoagulation monitoring, referral networks, obstetric and cardiac anesthesia access, and integrated. In review

pregnancy heart teams capable of delivering multidisciplinary care throughout pregnancy and the postpartum period. Importantly, RHD elucidates a broader principle in global cardiovascular health: the burden of disease is not solely determined by pathophysiology but by structural inequities in healthcare access and delivery. As global initiatives increasingly prioritize cardiovascular disease within maternal health agendas, developing effective strategies that extend beyond clinical management to encompass prevention, early detection, and system-level strengthening are warranted. Addressing these covariate determinants offers an opportunity to reduce the disproportionate burden of RHD-related pregnancy complications and advance progress toward equitable maternal cardiovascular care on a global scale.
