# Supplementary figures and images for: Global disparities in the detection and management of rheumatic heart disease in pregnancy across endemic settings
Source: Front Surg. 2026 Jul 15;13:1838220. doi: 10.3389/fsurg.2026.1838220 (PMC13417632; doi:10.3389/fsurg.2026.1838220)

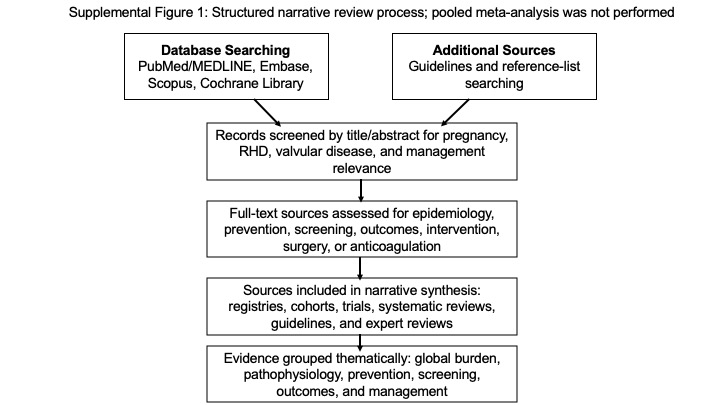

Supplement: Supplementary file 1 [file Image1.jpeg]
